# Tetra­potassium *cis*-dioxido-*trans*-bis­(sulfato-κ*O*)sulfato(κ^2^ *O*,*O*′)molybdate(VI)

**DOI:** 10.1107/S1600536808003851

**Published:** 2008-02-13

**Authors:** Susan J. Cline Schäffer, Rolf W. Berg

**Affiliations:** a The Technical University of Denmark, Department of Chemistry, Building 207, DK-2800 Lyngby, Denmark

## Abstract

The title compound, K_4_[Mo^VI^O_2_(SO_4_)_3_], was precipitated from a melt of molybdenum(VI) oxide and potassium sulfate in potassium disulfate. The compound contains monomeric [Mo^VI^O_2_(SO_4_)_3_]^4−^ anions, with the Mo^VI^ atom, both oxide ligands, and the S atom and both ligating O atoms of the bidentate sulfate group lying on a crystallographic mirror plane. One of the potassium cations is nine-coordinate, while the other is eight-coordinate.

## Related literature

For related literature, see: Topsøe & Nielsen (1947 ▶); Berg & Thorup (2005 ▶); Borup *et al.* (1990 ▶); Nørbygaard *et al.* (1998 ▶); Nielsen *et al.* (1993 ▶); Rasmussen *et al.* (2003 ▶); Salles *et al.* (1996 ▶); Schäffer & Berg (2005 ▶); Tamasi & Cini (2003 ▶).
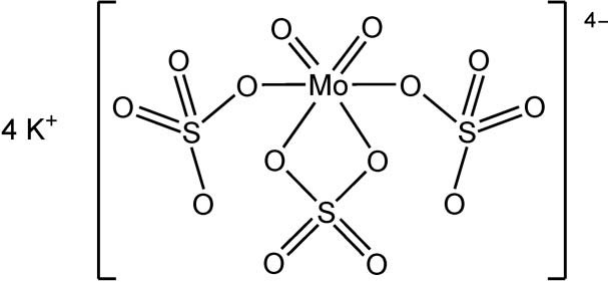


## Experimental

### 

#### Crystal data

K_4_[MoO_2_(SO_4_)_3_]
*M* *_r_* = 572.52
Orthorhombic, 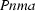

*a* = 7.5931 (5) Å
*b* = 17.1276 (11) Å
*c* = 10.5132 (7) Å
*V* = 1367.26 (16) Å^3^
*Z* = 4
Mo *K*α radiation
μ = 2.71 mm^−1^
*T* = 120 (2) K
0.28 × 0.18 × 0.07 mm

#### Data collection

Bruker SMART APEX CCD diffractometer
Absorption correction: Gaussian (*XPREP*; Bruker, 2002 ▶) *T*_min_ = 0.512, *T*_max_ = 0.684
16179 measured reflections
1693 independent reflections
1681 reflections with *I* > 2σ(*I*)
*R*_int_ = 0.018

#### Refinement

*R*[*F* ^2^ > 2σ(*F* ^2^)] = 0.016
*wR*(*F* ^2^) = 0.041
*S* = 1.11
1693 reflections
110 parameters
Δρ_max_ = 0.59 e Å^−3^
Δρ_min_ = −0.46 e Å^−3^

### 

Data collection: *SMART* (Bruker, 2002 ▶); cell refinement: *SAINT* (Bruker, 2002 ▶); data reduction: *SAINT*; program(s) used to solve structure: *SHELXTL* (Sheldrick, 2008 ▶); program(s) used to refine structure: *SHELXTL*; molecular graphics: *SHELXTL*; software used to prepare material for publication: *SHELXTL*.

## Supplementary Material

Crystal structure: contains datablocks global, I. DOI: 10.1107/S1600536808003851/bi2277sup1.cif

Structure factors: contains datablocks I. DOI: 10.1107/S1600536808003851/bi2277Isup2.hkl

Additional supplementary materials:  crystallographic information; 3D view; checkCIF report

## Figures and Tables

**Table:** 

Mo1—O1	1.6889 (18)
Mo1—O2	1.6883 (18)
Mo1—O3	2.2665 (17)
Mo1—O4	2.1837 (16)
Mo1—O6	2.0365 (12)
K1—O5^i^	2.6408 (12)
K1—O8^ii^	3.2305 (14)
K2—O8^iii^	2.7005 (14)
K2—O6^iv^	3.0239 (13)

**Table:** 

O2—Mo1—O1	105.77 (10)
O2—Mo1—O6	98.04 (4)
O1—Mo1—O6	94.84 (4)
O6—Mo1—O6^v^	158.22 (7)
O2—Mo1—O4	155.71 (8)
O1—Mo1—O4	98.52 (8)
O6—Mo1—O4	79.65 (3)
O2—Mo1—O3	92.29 (8)
O1—Mo1—O3	161.94 (8)
O6—Mo1—O3	82.38 (3)
O4—Mo1—O3	63.43 (6)

Symmetry codes: (i) 
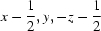
; (ii) 
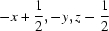
; (iii) 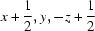; (iv) 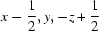; (v) 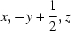.

**Fig. 1.**
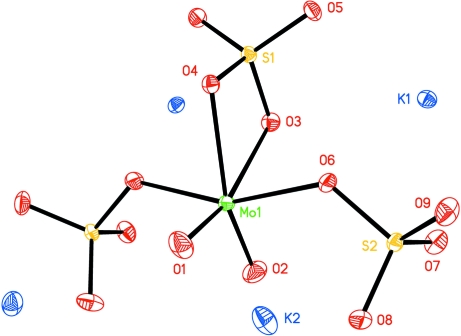
The [MoVIO2(SO4)3]4- anion and four K+ cations, showing displacement ellipsoids at 50% probability.

**Fig. 2.**
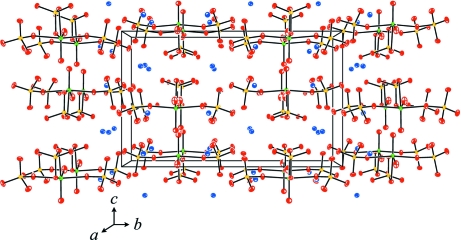
The crystal packing, viewed along the a axis.

## supplementary crystallographic information

### Comment

Efforts to improve the industrial vanadium-based sulfuric acid catalyst (Topsøe & Nielsen, 1947) have led to investigations of potassium disulfate's use as a suitable solvent for the production of new sulfate-containing catalysts. Many of the previously reported sulfato compounds precipitated from melts of potassium disulfate contain polymeric anions (Nørbygaard *et al.*, 1998, Berg & Thorup, 2005), while some contain dimers (Nielsen *et al.*, 1993, Rasmussen *et al.*, 2003, Schäffer & Berg, 2005). Monomers such as that in the title compound (Fig. 1) are less common (Borup *et al.*, 1990).

The coordination sphere of the Mo^VI^ atom contains two oxido ligands, two terminally bound sulfato ligands, and a bidentate sulfato ligand. Because of the bidentate ligand, the coordination sphere angles (see Table 1) in the mirror plane are grossly distorted from octahedral. The O3—Mo1—O4 angle is only 63.43 (6)°, while the O1—Mo1—O2 angle opposite to it is 105.77 (10)°. Deviations of 10° or less are found for the other angles between *cis* O atoms. The Mo=O bond distances are nearly equivalent (1.6883 (18)Å & 1.6889 (18) Å), which is expected since both bonds are *trans* to O atoms in the bidentate sulfato ligand. The Mo—O distance to the terminal sulfato [Mo1—O6] is slightly shorter than those to the bidentate sulfato ligand [Mo1—O3 and Mo1—O4]. The Mo—O distances compare well with previously reported values (Salles *et al.*, 1996, Nørbygaard *et al.*, 1998).

The S—O bond distances of 1.5044 (17)–1.5532 (17)Å involving O bound to Mo are longer than the terminal S—O bond lengths of 1.4497–1.4621 (12) Å, an effect typical for sulfato complexes of many different transition metal centers (Borup *et al.*, 1990, Nielsen *et al.*, 1993, Berg & Thorup, 2005). The sulfato ligands have approximately tetrahedral geometry. The angles vary from 101.14° for O3—S1—O4 to 114.68 (10)° for O5—S1—O5^i^ [symmetry code (i): *x*, 1/2 - *y*, *z*]; both extremes involve the bidentate sulfato ligand. Five of the eight remaining independent O—S—O angles deviate by less than 2° from ideal. The potassium cation, K1, is nine-coordinate, while K2 is eight-coordinate. The K—O distances range from 2.6408 to 3.2305 (14) Å.

### Experimental

Crystals were grown from a melt of 28.0 mol% molybdenum(VI) oxide, 43.9 mol% potassium sulfate, and 28.1 mol% potassium disulfate, using a method described previously (Nørbygaard *et al.*, 1998).

### Refinement

(type here to add refinement details)

### Crystal data

**Table:**

K_4_[MoO_2_(SO_4_)_3_]	*F*_000_ = 1112
*M**_r_* = 572.52	*D*_x_ = 2.781 Mg m^−^^3^
Orthorhombic, *P**n**m**a*	Mo *K*α radiation λ = 0.71073 Å
Hall symbol: -P 2ac 2n	Cell parameters from 9999 reflections
*a* = 7.5931 (5) Å	θ = 2.3–28.0º
*b* = 17.1276 (11) Å	µ = 2.71 mm^−^^1^
*c* = 10.5132 (7) Å	*T* = 120 (2) K
*V* = 1367.26 (16) Å^3^	Tabular, colorless
*Z* = 4	0.28 × 0.18 × 0.08 mm

### Data collection

**Table:**

Bruker SMART APEX CCD diffractometer	1693 independent reflections
Radiation source: normal-focus sealed tube	1681 reflections with *I* > 2σ(*I*)
Monochromator: graphite	*R*_int_ = 0.019
*T* = 120(2) K	θ_max_ = 28.0º
ω scans	θ_min_ = 2.3º
Absorption correction: gaussian(XPREP; Bruker, 2002)	*h* = −9→10
*T*_min_ = 0.512, *T*_max_ = 0.684	*k* = −22→22
16179 measured reflections	*l* = −13→13

### Refinement

**Table:**

Refinement on *F*^2^	Secondary atom site location: difference Fourier map
Least-squares matrix: full	*w* = 1/[σ^2^(*F*_o_^2^) + (0.0174*P*)^2^ + 1.7854*P*] where *P* = (*F*_o_^2^ + 2*F*_c_^2^)/3
*R*[*F*^2^ > 2σ(*F*^2^)] = 0.016	(Δ/σ)_max_ < 0.001
*wR*(*F*^2^) = 0.041	Δρ_max_ = 0.59 e Å^−^^3^
*S* = 1.11	Δρ_min_ = −0.46 e Å^−^^3^
1693 reflections	Extinction correction: SHELXTL (Sheldrick, 2008), Fc^*^=kFc[1+0.001xFc^2^λ^3^/sin(2θ)]^-1/4^
110 parameters	Extinction coefficient: 0.00164 (15)
Primary atom site location: structure-invariant direct methods	

### Special details

**Table:**

Experimental. Five series of frames were filtered for statistical outliers then corrected for absorption using *XPREP* in *SHELXTL* (Sheldrick, 2008) before using *SAINT* & *SADABS* (Bruker, 2002) to sort, merge, and scale the combined data. A series of identical frames was collected twice during the experiment to monitor decay, and none was observed.
Geometry. All e.s.d.'s (except the e.s.d. in the dihedral angle between two l.s. planes) are estimated using the full covariance matrix. The cell e.s.d.'s are taken into account individually in the estimation of e.s.d.'s in distances, angles and torsion angles; correlations between e.s.d.'s in cell parameters are only used when they are defined by crystal symmetry. An approximate (isotropic) treatment of cell e.s.d.'s is used for estimating e.s.d.'s involving l.s. planes.
Refinement. Refinement of *F*^2^ against ALL reflections. The weighted *R*-factor *wR* and goodness of fit *S* are based on *F*^2^, conventional *R*-factors *R* are based on *F*, with *F* set to zero for negative *F*^2^. The threshold expression of *F*^2^ > σ(*F*^2^) is used only for calculating *R*-factors(gt) *etc*. and is not relevant to the choice of reflections for refinement. *R*-factors based on *F*^2^ are statistically about twice as large as those based on *F*, and *R*- factors based on ALL data will be even larger.

### Fractional atomic coordinates and isotropic or equivalent isotropic displacement parameters (Å^2^)

**Table:**

	*x*	*y*	*z*	*U*_iso_*/*U*_eq_	
K1	0.43682 (4)	0.08865 (2)	−0.24076 (3)	0.01208 (8)	
K2	0.25655 (5)	0.10844 (2)	0.39352 (3)	0.01644 (9)	
Mo1	0.32312 (3)	0.2500	0.065899 (18)	0.00961 (7)	
S1	0.62301 (7)	0.2500	−0.09790 (5)	0.00926 (11)	
S2	0.23382 (5)	0.06574 (2)	0.06161 (3)	0.00949 (9)	
O1	0.3105 (3)	0.2500	0.22628 (17)	0.0209 (4)	
O2	0.1129 (2)	0.2500	0.0135 (2)	0.0219 (4)	
O3	0.4316 (2)	0.2500	−0.13493 (15)	0.0124 (3)	
O4	0.6095 (2)	0.2500	0.04687 (15)	0.0102 (3)	
O5	0.70888 (16)	0.17874 (7)	−0.13903 (11)	0.0135 (2)	
O6	0.36968 (15)	0.13324 (7)	0.05145 (11)	0.0134 (2)	
O7	0.16034 (17)	0.05557 (8)	−0.06586 (11)	0.0161 (3)	
O8	0.10253 (17)	0.08813 (8)	0.15593 (12)	0.0184 (3)	
O9	0.33322 (16)	−0.00276 (7)	0.10186 (13)	0.0176 (3)	

### Atomic displacement parameters (Å^2^)

**Table:**

	*U*^11^	*U*^22^	*U*^33^	*U*^12^	*U*^13^	*U*^23^
K1	0.01082 (17)	0.01361 (16)	0.01182 (16)	0.00149 (12)	−0.00089 (12)	0.00023 (12)
K2	0.02176 (19)	0.01335 (17)	0.01422 (17)	0.00508 (14)	0.00204 (14)	−0.00023 (13)
Mo1	0.00891 (10)	0.00789 (10)	0.01203 (11)	0.000	0.00220 (7)	0.000
S1	0.0105 (2)	0.0077 (2)	0.0096 (2)	0.000	0.0013 (2)	0.000
S2	0.01081 (18)	0.00829 (17)	0.00938 (18)	−0.00132 (14)	0.00002 (13)	0.00071 (13)
O1	0.0304 (11)	0.0172 (9)	0.0150 (9)	0.000	0.0080 (8)	0.000
O2	0.0117 (8)	0.0178 (9)	0.0364 (11)	0.000	−0.0020 (8)	0.000
O3	0.0119 (8)	0.0139 (8)	0.0115 (7)	0.000	−0.0019 (6)	0.000
O4	0.0115 (8)	0.0102 (7)	0.0089 (7)	0.000	0.0001 (6)	0.000
O5	0.0158 (6)	0.0095 (5)	0.0153 (6)	0.0017 (4)	0.0044 (5)	−0.0012 (4)
O6	0.0116 (5)	0.0088 (5)	0.0197 (6)	−0.0019 (4)	0.0022 (5)	−0.0001 (4)
O7	0.0187 (6)	0.0186 (6)	0.0111 (6)	−0.0037 (5)	−0.0034 (4)	0.0017 (4)
O8	0.0163 (6)	0.0201 (6)	0.0187 (6)	−0.0045 (5)	0.0072 (5)	−0.0055 (5)
O9	0.0181 (6)	0.0116 (6)	0.0232 (6)	−0.0002 (5)	−0.0046 (5)	0.0058 (5)

### Geometric parameters (Å, °)

**Table:**

Mo1—O1	1.6889 (18)	K2—O8^vi^	2.7005 (14)
Mo1—O2	1.6883 (18)	K2—O4^vii^	2.7419 (8)
Mo1—O3	2.2665 (17)	K2—O8	2.7799 (14)
Mo1—O4	2.1837 (16)	K2—O5^vii^	2.8710 (13)
Mo1—O6	2.0365 (12)	K2—O7^viii^	2.9106 (14)
Mo1—O6^i^	2.0365 (12)	K2—O9^viii^	2.9220 (14)
S1—O3	1.5044 (17)	K2—O1	3.0229 (11)
S1—O4	1.5254 (16)	K2—O6^vii^	3.0239 (13)
S1—O5	1.4497 (12)	O1—K2^i^	3.0229 (11)
S1—O5^i^	1.4497 (12)	O3—K1^i^	2.9794 (7)
S2—O6	1.5532 (12)	O4—K2^ix^	2.7419 (8)
S2—O7	1.4621 (12)	O4—K2^vi^	2.7419 (8)
S2—O8	1.4575 (13)	O5—K1^iii^	2.6408 (12)
S2—O9	1.4577 (12)	O5—K2^vi^	2.8710 (13)
K1—O5^ii^	2.6408 (12)	O6—K2^vi^	3.0239 (13)
K1—O7^iii^	2.7083 (12)	O7—K1^ii^	2.7083 (12)
K1—O9^iv^	2.7102 (12)	O7—K2^v^	2.9106 (14)
K1—O5	2.7915 (13)	O8—K2^vii^	2.7005 (14)
K1—O7	2.8476 (13)	O8—K1^viii^	3.2305 (14)
K1—O3	2.9794 (7)	O9—K1^iv^	2.7102 (12)
K1—O9^v^	3.0176 (13)	O9—K2^v^	2.9220 (13)
K1—O6	3.2064 (12)	O9—K1^viii^	3.0176 (13)
K1—O8^v^	3.2305 (14)		
			
O5^ii^—K1—O7^iii^	100.00 (4)	O1—Mo1—O6^i^	94.84 (4)
O5^ii^—K1—O9^iv^	175.76 (4)	O6—Mo1—O6^i^	158.22 (7)
O7^iii^—K1—O9^iv^	83.52 (4)	O2—Mo1—O4	155.71 (8)
O5^ii^—K1—O5	110.20 (4)	O1—Mo1—O4	98.52 (8)
O7^iii^—K1—O5	86.53 (4)	O6—Mo1—O4	79.65 (3)
O9^iv^—K1—O5	67.47 (4)	O6^i^—Mo1—O4	79.65 (3)
O5^ii^—K1—O7	86.68 (4)	O2—Mo1—O3	92.29 (8)
O7^iii^—K1—O7	154.84 (5)	O1—Mo1—O3	161.94 (8)
O9^iv^—K1—O7	91.09 (4)	O6—Mo1—O3	82.38 (3)
O5—K1—O7	114.09 (4)	O6^i^—Mo1—O3	82.38 (3)
O5^ii^—K1—O3	68.16 (4)	O4—Mo1—O3	63.43 (6)
O7^iii^—K1—O3	118.86 (4)	O3—S1—O4	101.14 (9)
O9^iv^—K1—O3	108.11 (4)	O5—S1—O5^i^	114.68 (10)
O5—K1—O3	49.76 (4)	O5—S1—O3	110.93 (6)
O7—K1—O3	86.20 (4)	O5^i^—S1—O3	110.93 (6)
O5^ii^—K1—O9^v^	64.98 (4)	O5—S1—O4	109.14 (6)
O7^iii^—K1—O9^v^	84.97 (4)	O5^i^—S1—O4	109.14 (6)
O9^iv^—K1—O9^v^	117.94 (5)	O8—S2—O9	111.62 (8)
O5—K1—O9^v^	169.23 (4)	O8—S2—O7	113.20 (8)
O7—K1—O9^v^	75.89 (4)	O9—S2—O7	111.58 (8)
O3—K1—O9^v^	130.39 (4)	O8—S2—O6	107.77 (7)
O5^ii^—K1—O6	102.42 (4)	O9—S2—O6	105.97 (7)
O7^iii^—K1—O6	150.31 (4)	O7—S2—O6	106.20 (7)
O9^iv^—K1—O6	73.47 (4)	Mo1—O1—K2^i^	126.04 (3)
O5—K1—O6	67.60 (3)	Mo1—O1—K2	126.04 (3)
O7—K1—O6	46.49 (3)	K2^i^—O1—K2	106.66 (6)
O3—K1—O6	54.49 (4)	S1—O3—Mo1	96.32 (8)
O9^v^—K1—O6	122.24 (3)	S1—O3—K1	94.81 (4)
O5^ii^—K1—O8^v^	109.03 (4)	Mo1—O3—K1	110.65 (3)
O7^iii^—K1—O8^v^	67.02 (4)	S1—O3—K1^i^	94.81 (4)
O9^iv^—K1—O8^v^	74.46 (4)	Mo1—O3—K1^i^	110.65 (3)
O5—K1—O8^v^	135.67 (3)	K1—O3—K1^i^	136.12 (6)
O7—K1—O8^v^	87.85 (4)	S1—O4—Mo1	99.11 (8)
O3—K1—O8^v^	173.55 (4)	S1—O4—K2^ix^	101.58 (4)
O9^v^—K1—O8^v^	45.24 (3)	Mo1—O4—K2^ix^	112.62 (4)
O6—K1—O8^v^	122.05 (3)	S1—O4—K2^vi^	101.58 (4)
O8^vi^—K2—O4^vii^	123.65 (4)	Mo1—O4—K2^vi^	112.62 (4)
O8^vi^—K2—O8	102.72 (3)	K2^ix^—O4—K2^vi^	124.32 (6)
O4^vii^—K2—O8	98.32 (4)	S1—O5—K1^iii^	158.35 (7)
O8^vi^—K2—O5^vii^	110.47 (4)	S1—O5—K1	104.29 (6)
O4^vii^—K2—O5^vii^	51.12 (4)	K1^iii^—O5—K1	88.78 (4)
O8—K2—O5^vii^	143.83 (4)	S1—O5—K2^vi^	98.14 (6)
O8^vi^—K2—O7^viii^	72.12 (4)	K1^iii^—O5—K2^vi^	95.88 (4)
O4^vii^—K2—O7^viii^	155.26 (4)	K1—O5—K2^vi^	101.88 (4)
O8—K2—O7^viii^	95.87 (4)	S2—O6—Mo1	127.63 (7)
O5^vii^—K2—O7^viii^	107.47 (3)	S2—O6—K2^vi^	121.83 (6)
O8^vi^—K2—O9^viii^	106.95 (4)	Mo1—O6—K2^vi^	107.00 (5)
O4^vii^—K2—O9^viii^	106.35 (4)	S2—O6—K1	89.66 (5)
O8—K2—O9^viii^	119.86 (4)	Mo1—O6—K1	109.45 (5)
O5^vii^—K2—O9^viii^	63.67 (3)	K2^vi^—O6—K1	89.74 (3)
O7^viii^—K2—O9^viii^	48.91 (3)	S2—O7—K1^ii^	154.47 (8)
O8^vi^—K2—O1	81.93 (5)	S2—O7—K1	106.67 (6)
O4^vii^—K2—O1	58.59 (4)	K1^ii^—O7—K1	86.31 (3)
O8—K2—O1	68.58 (4)	S2—O7—K2^v^	99.59 (6)
O5^vii^—K2—O1	101.75 (4)	K1^ii^—O7—K2^v^	103.16 (4)
O7^viii^—K2—O1	146.11 (4)	K1—O7—K2^v^	86.41 (4)
O9^viii^—K2—O1	164.67 (4)	S2—O8—K2^vii^	124.63 (7)
O8^vi^—K2—O6^vii^	179.32 (4)	S2—O8—K2	110.89 (7)
O4^vii^—K2—O6^vii^	55.70 (4)	K2^vii^—O8—K2	124.48 (5)
O8—K2—O6^vii^	77.34 (4)	S2—O8—K1^viii^	92.60 (6)
O5^vii^—K2—O6^vii^	69.31 (3)	K2^vii^—O8—K1^viii^	95.48 (4)
O7^viii^—K2—O6^vii^	108.55 (3)	K2—O8—K1^viii^	81.61 (4)
O9^viii^—K2—O6^vii^	73.56 (3)	S2—O9—K1^iv^	157.94 (8)
O1—K2—O6^vii^	97.48 (4)	S2—O9—K2^v^	99.22 (6)
O2—Mo1—O1	105.77 (10)	K1^iv^—O9—K2^v^	102.59 (4)
O2—Mo1—O6	98.04 (4)	S2—O9—K1^viii^	101.52 (6)
O1—Mo1—O6	94.84 (4)	K1^iv^—O9—K1^viii^	82.98 (3)
O2—Mo1—O6^i^	98.04 (4)	K2^v^—O9—K1^viii^	87.16 (3)

Symmetry codes: (i) *x*, −*y*+1/2, *z*; (ii) *x*−1/2, *y*, −*z*−1/2; (iii) *x*+1/2, *y*, −*z*−1/2; (iv) −*x*+1, −*y*, −*z*; (v) −*x*+1/2, −*y*, *z*−1/2; (vi) *x*+1/2, *y*, −*z*+1/2; (vii) *x*−1/2, *y*, −*z*+1/2; (viii) −*x*+1/2, −*y*, *z*+1/2; (ix) *x*+1/2, −*y*+1/2, −*z*+1/2.
